# The Mare Model to Study the Effects of Ovarian Dynamics on Preantral Follicle Features

**DOI:** 10.1371/journal.pone.0149693

**Published:** 2016-02-22

**Authors:** Kele A. Alves, Benner G. Alves, Gustavo D. A. Gastal, Saulo G. S. de Tarso, Melba O. Gastal, José R. Figueiredo, Maria L. Gambarini, Eduardo L. Gastal

**Affiliations:** 1 Department of Animal Science, Food and Nutrition, Southern Illinois University, Carbondale, lllinois, United States of America; 2 Center for Studies and Research in Animal Reproductive Biology, College of Veterinary and Animal Science, Federal University of Goiás, Goiânia, GO, Brazil; 3 Laboratory of Manipulation of Oocytes and Preantral Follicles (LAMOFOPA), Faculty of Veterinary Medicine, State University of Ceará, Fortaleza, CE, Brazil; Faculty of Animal Sciences and Food Engineering, University of São Paulo, BRAZIL

## Abstract

Ovarian tissue collected by biopsy procedures allows the performance of many studies with clinical applications in the field of female fertility preservation. The aim of the present study was to investigate the influence of reproductive phase (anestrous vs. diestrous) and ovarian structures (antral follicles and corpus luteum) on the quality, class distribution, number, and density of preantral follicles, and stromal cell density. Ovarian fragments were harvested by biopsy pick-up procedures from mares and submitted to histological analysis. The mean preantral follicle and ovarian stromal cell densities were greater in the diestrous phase and a positive correlation of stromal cell density with the number and density of preantral follicles was observed. The mean area (mm^2^) of ovarian structures increased in the diestrous phase and had positive correlations with number of preantral follicles, follicle density, and stromal cell density. Biopsy fragments collected from ovaries containing an active corpus luteum had a higher follicle density, stromal cell density, and proportion of normal preantral follicles. In conclusion, our results showed: (1) the diestrous phase influenced positively the preantral follicle quality, class distribution, and follicle and stromal cell densities; (2) the area of ovarian structures was positively correlated with the follicle and stromal cell densities; and (3) the presence of an active corpus luteum had a positive effect on the quality of preantral follicles, and follicle and stromal densities. Therefore, herein we demonstrate that the presence of key ovarian structures favors the harvest of ovarian fragments containing an appropriate number of healthy preantral follicles.

## Introduction

The mare has been advocated by many researchers as an appropriate comparative animal model to study antral follicular dynamics in women [1–9] due to some similarities in reproductive events (e.g., follicular waves, hormonal concentration changes, age-related decline in fertility, acyclic conditions, and anovulatory dysfunctions such as cysts or luteinized unruptured follicles/hemorrhagic anovulatory follicles). Women and mares are monovular species and although the seasonality does not occur in women, this characteristic in an animal model allows the evaluation of which internal ovarian factors may be operating when acyclic patterns are occurring [3]. More recently, the mare has also been suggested as a model for studies related to preantral follicles [10–13]. Therefore, the search for an appropriate animal model for comparative studies of preantral follicle population, density, and distribution has been a major focus of ovarian translational studies [14].

The preantral follicle population represents the finite and available reserve of gametes that can be recruited during the reproductive lifespan. In order to explore such potential, it is necessary to understand some anatomical-physiological aspects related to the ovary. The follicular heterogeneity observed in ovarian fragments of different species (mares: [10]; women: [15]; cows: [16]; and ewes: [17]) limits the harvesting and more widespread use of preantral follicles in assisted reproductive technologies (ARTs), such as *in vitro* follicle culture, and ovarian cryopreservation and transplant. The ovarian parenchyma contains a considerable area of stroma which suffers profound structural changes during the different reproductive phases due to the dynamics of antral follicles and corpus luteum. Furthermore, stromal cell density is an important indicator of tissue integrity, plays a role in follicle development [18–20], and is considered an important parameter for maintaining the functionality of preantral follicles. Previous studies reported that ovarian structures produce hormones and growth factors that can stimulate the development and viability of preantral follicles [21–25]. In addition, cellular mechanochemical processes and changes in the extracellular matrix govern tissue morphogenesis during different reproductive phases [26]. Therefore, studies evaluating the influence of ovarian parenchymal structures such as antral follicles (tertiary and preovulatory follicles) and corpus luteum on the number, density, and quality of preantral follicles are needed.

Ovarian biopsy has been an important tool used to harvest preantral follicles for use in clinical and research settings, allowing female fertility preservation in several species (mares: [11, 12]; women: [15, 27, 28, 29]; and cows: [16, 30]). This safe method ensures the collection of preantral follicles without jeopardizing the individual’s reproductive life [10, 31]. To the best of our knowledge, there are no studies in horses reporting established guidelines to identify the most suitable reproductive phase (e.g., diestrous vs. anestrous) to perform ovarian biopsy procedures for harvesting a suitable number of healthy preantral follicles associated with higher follicular and stromal cell densities.

The aim of this study was to assess the influence of reproductive phases (anestrous and diestrous) and ovarian structures (antral follicles and corpus luteum) on the: (1) quality, class distribution, number, and density of preantral follicles, and (2) stromal cell density in ovarian fragments harvested by biopsy procedure.

## Materials and Methods

### Animals

All experimental procedures were performed according to the United States Government Principles for the Utilization and Care of Vertebrate Animals Used in Testing, Research and Training. The research protocol (#11–007) was approved by the Institutional Animal Care and Use Committee of Southern Illinois University. The study was carried out during the winter (December to March) and spring seasons (April to June) in the northern hemisphere. Light horse mares (n = 10) that weighed between 400 and 600 Kg and were 5 to 11 years old were used. No hormonal treatments were administered during the experimental period. Mares were kept on pasture and orchard grass/alfalfa mixed hay, supplemented with balanced grain ration and minerals.

### Area of ovarian structures

The ovaries and uterus of each mare were scanned every other day for two months during the winter and spring seasons using a transrectal ultrasound scanner (Aloka SSD-900, Aloka Co., LTD., Wallingford, CT, USA) equipped with a 5 to 10 MHz linear array transducer (Aloka UST-5821-7.5). At the moment of the biopsy procedure, the ovarian structures were recorded as small follicles (6 to 26 mm of diameter), preovulatory follicles (≥36 mm), and corpus luteum (≥30 mm, days 4 to 12 of estrous cycle). Therefore, the following classifications were generated: a) small follicles (ovary with only small follicles); b) corpus luteum (ovary with small follicles and a corpus luteum); and c) preovulatory follicle (ovary with small follicles and a preovulatory follicle). The height and width of the ovarian structures were recorded at the maximal length using the caliper of the ultrasound scanner software and the average of these measures was used to determine each structure’s diameter [32]. Thereafter, the area (mm^2^) of each ovarian structure was calculated by the following formula: Area = π × (D/2)^2^; where: π = 3.14; D = diameter (mm).

### Ovarian tissue collection

Biopsy fragments (n = 3 to 4) were collected from each ovary (left and right) separately via the biopsy pick-up technique [10]. Briefly, before each biopsy procedure, analgesia (flunixin meglumine; Flunixiject, 1.1 mg/kg iv; Butler Schein Animal Health, Dublin, OH, USA), rectal relaxation (hyoscine N-butyl bromide; Buscopan, 0.2 mg/kg iv), and sedation (xylazine; AnaSed, 1 mg/kg iv; Lloyd Laboratories, Shenandoah, IA, USA and buthorphanol tartrate; Dolorex, 0.05 mg/kg iv; Intervet / Shering-Plough Animal Health, Millsboro, DE, USA) were induced. Mares were administered penicillin (Agri-Cillin, 6500 U/kg im; AgriLabs, ST. Joseph, MO, USA) immediately after each procedure and for the next two days. The BPU device used was a 48 cm-long, automated, spring-loaded instrument with an inner trocar point plunger containing a 15 × 1.6 mm specimen notch surrounded by an outer 16 ga cutting needle (US Biopsy, Franklin, IN, USA). This device was introduced through a needle guide mounted on a probe handle with a 5 to 10 MHz transvaginal ultrasound-guided convex array transducer (Aloka UST-987-7.5), which was used for placement of the biopsy needle within the ovary. The ovary was manipulated transrectally and positioned against the vaginal wall so that the projected needle path could be visualized. When the needle was properly positioned in the ovary, the inner stylet was advanced to expose the specimen notch. The spring-loaded device was then fired, which propelled the cutting cannula over the specimen notch, thus collecting any ovarian tissue resting within the notch. The Biopsy Pick-Up (BPU) needle was then removed from the transvaginal ultrasound extension probe and the specimen notch exposed in order to retrieve the biopsy fragment. All procedures were performed by the same operator. The ultrasound-guided BPU is a non-invasive method that allows the repeatedly harvesting of ovarian tissue fragments containing large numbers of preantral follicles from living mares without affecting their normal short-term ovarian function or general reproductive health [10, 11, 12].

The procedures were performed during the winter (anestrous phase; follicles ≤20 mm, absence of corpus luteum, ovulation, and estrus signs) and spring seasons (diestrous phase; days 4 to 12 of estrous cycle, presence of a corpus luteum and compatible uterine echotexture). The same mares were used in both seasons.

### Histological processing and follicle density

Ovarian fragments were fixed in Bouin’s solution for 2 hours and then kept in 70% alcohol until histological preparation. The fragments were embedded in paraffin wax and totally cut into serial sections (7 μm). Every section was mounted and stained with acid Schiff (PAS) and hematoxylin. All slides were scanned and the perimeter of digital images from histological sections was delimited with a photo editing program (Adobe Photoshop CS4, San Jose, CA, USA) after a scale calibration, and the area’s measurement (cm^2^) was recorded. Thereafter, the follicle density was determined by the following formula: follicle density = number of preantral follicles/area of the ovarian section (cm^2^) as previously reported [13].

### Microscopy and end points

Histological sections were analyzed on a light microscope (Nikon E200, Tokyo, Japan) at magnification ×400 and an image capture system (LEICA Imaging Software, Wetzlar, Germany). The following end points were recorded: number of preantral follicles and follicle density per ovarian fragment, follicle class distribution, follicle morphology (normal and abnormal), and ovarian stromal cell density. The preantral follicles were classified according to their developmental stage into primordial, transitional, primary, and secondary, as previously described [10].

### Morphology of preantral follicles

Only preantral follicles with visualized oocyte nucleus were counted and morphologically classified as normal (follicle containing an intact oocyte and oocyte nucleus surrounded by granulosa cells well organized in one or more layers) or abnormal (follicles with a retracted cytoplasm or disorganized granulosa cell layers detached from the basement membrane and oocyte with pyknotic nucleus [33]).

### Ovarian stromal cell density

Ovarian stromal cell density was evaluated as described by Commin et al. [34], with some modifications. Briefly, a total of 10% of histological sections for each ovarian fragment were analyzed. Four random fields (50 × 50 μm = 2,500 μm^2^) were selected and the stromal cell nuclei were counted to calculate the mean of stromal cell density per ovarian fragment. All evaluations and measurements were performed by a single operator.

### Statistical analyses

All statistical analyses were performed using R statistical software version 3.0.2 (R Foundation for Statistical Computing, Vienna, Austria). Data for end points that were not normally distributed were transformed (base 10 logarithmic). The number of preantral follicles and follicle density per ovarian fragment within the same reproductive phase (anestrous or diestrous) and side ovary (left or right) were compared by Kruskal-Wallis test, and among reproductive phases and side ovary by Wilcoxon-Mann-Whitney test. The follicle class distribution and the percentage of normal preantral follicles among reproductive phases were analyzed by chi-square test and G-test. Wilcoxon-Mann-Whitney test was used to compare the stromal cell density and area of the ovarian structures among the reproductive phases and side ovary. In addition, the former test was used to analyze the number of preantral follicles, follicle density, and stromal cell density in biopsy fragments harvested from ovaries with antral follicles and corpus luteum. Correlations between the number of preantral follicles and follicle density with stromal cell density, and among the number of preantral follicles, follicle density and stromal cell density with the area of ovarian structures were estimated by Spearman correlation analysis. Data are presented as mean ± SEM, unless otherwise stated. A probability of *P* < 0.05 indicated that a difference was significant, and *P* > 0.05 and ≤ 0.1 indicated that a difference approached significance.

## Results

### Ovarian biopsy fragments

A total of 142 ovarian fragments were collected by the biopsy pick-up method (mean, 3.7 fragments per ovary in each reproductive phase) and 13,462 histological sections were evaluated. Overall, 1,493 preantral follicles were recorded with a mean of 10.5 ± 1.7 follicles per ovarian fragment [range, 0 to 165; mean coefficient of variation (CV) = 195%]. No adverse effects were observed in the mares’ cyclicity or general reproductive health after any procedure.

### Effect of reproductive phase on preantral follicle density

The mean follicle density was 2.7 follicles per cm^2^ (range, 0 to 39; CV = 214%) and differed (P < 0.05) among mares (Table 1). This parameter varied within the same mare in different reproductive phases and in the overall mean a higher (P < 0.05) follicle density was observed in the diestrous when compared with the anestrous phase. Data of follicle density related to the ovary side (left and right) in different reproductive phases are shown (Table 2). Regardless of reproductive phase, the overall mean follicle density was greater (P < 0.05) in the right ovary. Furthermore, there was an increase (P < 0.05) in the overall mean follicle density in the diestrous phase for both ovary sides.

**Table 1:** Mean (± SEM) density of equine preantral follicles in ovarian biopsy fragments collected during anestrous and diestrous phases.

	Follicle density (number of preantral follicles/cm^2^)		
Mare	Anestrous (n = 73)^†^	Diestrous (n = 69)	Total (n = 142)
1	0.6 ± 0.1^a^^A^	1.5 ± 0.2^b^^A^	1.0 ± 0.1^A^^C^
2	0.8 ± 0.1^a^^A^^B^	1.0 ± 0.1^b^^A^^B^	0.9 ± 0.1^A^^C^
5	0.6 ± 0.1^a^^A^	0.7 ± 0.3^a^^B^	0.7 ± 0.1^A^
8	17.3 ± 2.3^a^^D^	16.0 ± 0.9^b^^D^	16.4 ± 1.0^B^
9	1.5 ± 0.2^a^^A^^B^	1.1 ± 0.2^a^^A^^B^	1.3 ± 0.1^A^^C^
10	0.7 ± 0.1^A^^B^		0.7 ± 0.1^A^^C^
11	0.7 ± 0.1^a^^A^^B^	0.5 ± 0.2^a^^B^	0.7 ± 0.1^A^
13	2.6 ± 0.3^a^^C^^E^	3.0 ± 0.4^a^^C^	2.7 ± 0.2^C^^D^
21	3.1 ± 0.3^a^^C^	3.8 ± 0.4^a^^C^	3.5 ± 0.2^D^
32	1.3 ± 0.2^a^^B^^E^	0.9 ± 0.2^b^^A^^B^	1.1 ± 0.1^A^^C^
Overall mean	2.1 ± 0.1^a^	3.5 ± 0.1^b^	2.7 ± 0.1

^†^ Total number of ovarian fragments collected via ovarian biopsy pick-up method.

^a,b^ Within a row, values without a common superscript differed (P < 0.05).

^A,B,C,D,E^ Within a column, values without a common superscript differed (P < 0.05).

Mare #10 did not participate in the diestrous phase of the experiment.

**Table 2:** Mean (± SEM) density of equine preantral follicles in ovarian biopsy fragments collected from left and right ovaries in anestrous and diestrous phases.

	Follicle density (number of preantral follicles/cm^2^)			
	Anestrous		Diestrous	
Mare	Left ovary (n = 37)^†^	Right ovary (n = 36)	Left ovary (n = 36)	Right ovary (n = 33)
1	0.5 ± 0.1^a^^A^^C^	0.7 ± 0.2^a^^A^	1.2 ± 0.2^a^^A^	1.8 ± 0.3^b^^A^^D^
2	0.9 ± 0.1^a^^A^^C^	0.8 ± 0.2^a^^A^^C^	1.0 ± 0.2^a^^A^	1.1 ± 0.2^a^^A^^B^
5	1.0 ± 0.2^a^^A^^C^	0.2 ± 0.1^b^^A^	1.4 ± 0.5^a^^A^	0.04 ± 0.0^b^^B^
8	10.9 ± 1.5^a^^B^^D^	24.6 ± 4.6^a^^D^	16.4 ± 1.7^a^^C^	15.7 ± 1.1^a^^C^
9	0.3 ± 0.1^a^^A^	3.3 ± 0.5^b^^B^	0.5 ± 0.2^a^^A^	1.7 ± 0.3^b^^A^^D^
10	0.9 ± 0.2^a^^A^^C^	0.5 ± 0.1^a^^A^^C^		
11	0.7 ± 0.2^a^^A^^C^	0.7 ± 0.2^a^^A^^C^	0.3 ± 0.2^a^^A^	0.8 ± 0.3^a^^A^^B^
13	3.0 ± 0.4^a^^D^	2.3 ± 0.4^b^^B^^C^	4.2 ± 0.7^a^^B^	1.8 ± 0.4^b^^A^^D^
21	4.6 ± 0.5^a^^B^	1.4 ± 0.3^b^^A^^B^	4.1 ± 0.7^a^^B^	3.7 ± 0.6^a^^D^
32	1.3 ± 0.2^a^^C^	1.4 ± 0.3^a^^A^^B^	0.1 ± 0.0^a^^A^	2.3 ± 0.6^b^^A^
Overall mean	1.9 ± 0.1^W^	2.3 ± 0.2^X^	3.0 ± 0.2^Y^	3.9 ± 0.2^Z^

^†^ Total number of ovarian fragments collected via ovarian biopsy pick-up method.

^a,b^ Within a row and same reproductive phase (anestrous or diestrous), values without a common superscript differed (P < 0.05).

^A,B,C,D^ Within a column, values without a common superscript differed (P < 0.05).

^W,X,Y,Z^ Within a row, regardless of the reproductive phase and ovary side, values without a common superscript differed (P < 0.05).

Mare #10 did not participate in the diestrous phase of the experiment.

The mean number of preantral follicles recorded per ovarian fragment during the anestrous and diestrous phases is shown (Table 3). The overall mean was similar among reproductive phases (P > 0.05), and among mares within the same phase a higher variability was observed (P < 0.05). Regardless of reproductive phase, no difference (P > 0.05) was observed for the number of preantral follicles between the left and right ovaries, but once again there was heterogeneity among animals and ovaries of the same animal (Table 4).

**Table 3:** Mean (± SEM) number of equine preantral follicles in ovarian biopsy fragments collected during anestrous and diestrous phases.

	Number of preantral follicles per ovarian fragment		
Mare	Anestrous (n = 73)^†^	Diestrous (n = 69)	Total (n = 142)
1	2.7 ± 1.0^a^^A^	9.1 ± 5.3^a^^A^^C^^E^	5.7 ± 2.6^A^^E^
2	4.7 ± 2.9^a^^A^^C^	4.7 ± 1.2^a^^A^^E^	4.7 ± 1.5^A^^C^^E^
5	2.7 ± 0.9^a^^A^	1.7 ± 0.9^a^^B^	2.2 ± 0.6^A^^E^
8	31.0 ± 2.4^a^^B^	60.8 ± 19.6^a^^D^	50.9 ± 13.4^D^
9	7.2 ± 3.5^a^^A^^D^	4.6 ± 1.8^a^^A^^E^	5.8 ± 1.9^E^
10	4.1 ± 1.2^A^^D^		4.1 ± 1.2^A^^E^
11	2.3 ± 0.8^a^^A^	1.2 ± 0.8^a^^B^	1.8 ± 0.5^A^
13	14.2 ± 5.6^a^^B^^C^^D^	8.6 ± 3.8^a^^E^	11.4 ± 3.3^B^^C^^E^
21	22.3 ± 10.3^a^^B^^D^	16.0 ± 4.5^a^^C^^E^	18.7 ± 4.9^B^
32	8.2 ± 3.4^a^^A^^D^	3.2 ± 1.9^b^^A^^B^	5.7 ± 2.0^A^^E^
Overall mean	8.5 ± 1.4^a^	12.6 ± 3.1^a^	10.5 ± 1.7

^†^ Total number of ovarian fragments collected via ovarian biopsy pick-up method.

^a,b^ Within a row, values without a common superscript differed (P < 0.05).

^A,B,C,D,E^ Within a column, values without a common superscript differed (P < 0.05).

Mare #10 did not participate in the diestrous phase of the experiment.

**Table 4:** Mean (± SEM) number of equine preantral follicles in ovarian biopsy fragments collected from left and right ovaries in anestrous and diestrous phases.

	Number of preantral follicles per ovarian fragment			
	Anestrous		Diestrous	
Mare	Left ovary (n = 37)^†^	Right ovary (n = 36)	Left ovary (n = 36)	Right ovary (n = 33)
1	2.5 ± 1.8	3.0 ± 1.2	5.2 ± 2.5	14.3 ± 12.8
2	6.7 ± 6.0	2.7 ± 0.6	5.0 ± 1.8	4.5 ± 1.8
5	4.2 ± 1.6	1.2 ± 0.6	2.7 ± 1.6	0.3 ± 0.3
8	29.5 ± 5.5	32.5 ± 1.5	44.0 ± 26.4	77.7 ± 30.1
9	1.7 ± 0.7	14.6 ± 6.4	1.7 ± 0.2	7.5 ± 3.2
10	3.7 ± 1.3	4.5 ± 2.2		
11	2.2 ± 1.2	2.5 ± 1.2	0.7 ± 0.4	2.0 ± 2.0
13	17.5 ± 10.2	11.0 ± 5.9	11.2 ± 7.6	6.0 ± 2.2
21	34.6 ± 18.2	10.0 ± 7.5	12.0 ± 5.2	20.0 ± 7.5
32	9.2 ± 4.9	7.2 ± 5.5	1.2 ± 1.2	5.2 ± 3.6
Overall mean^§^	9.5 ± 2.4	7.4 ± 1.6	9.3 ± 3.5	16.1 ± 5.4

^†^ Total number of ovarian fragments collected via ovarian biopsy pick-up method.

^§^ No difference (P > 0.05) was detected between ovaries within each reproductive phase.

Comparisons between ovaries and among mares within the same ovary and reproductive phase were not done because of the low number of observations (range, 2 to 4 fragments per ovary).

Mare #10 did not participate in the diestrous phase of the experiment.

### Effect of reproductive phase on preantral follicle class distribution and quality

**Fig 1:**
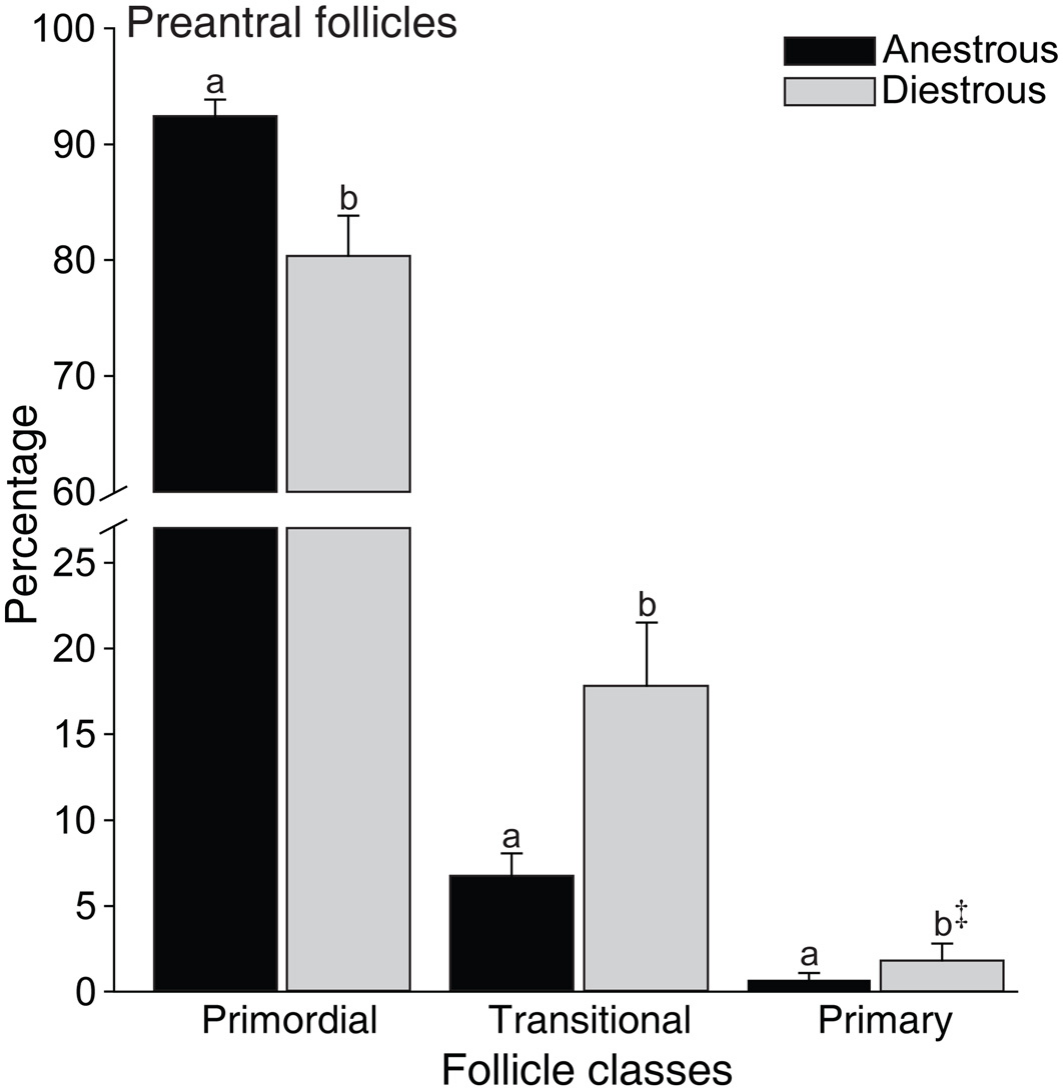
Mean (± SEM) percentage of equine preantral follicles according to class distribution in ovarian biopsy fragments collected during anestrous and diestrous phases. ^a,b^ Within the same follicle class, values without a common letter differed (*P* < 0.05). ^‡^ Tended to differ from transitional follicles during the same phase (*P* < 0.09).

The distribution of preantral follicle population was 85.3% primordial, 13.1% transitional, 1.4% primary, and 0.13% secondary. The percentage of primordial follicles was higher (P < 0.05) in the anestrous compared to the diestrous phase. In contrast, the proportion of growing follicles (transitional, P < 0.05; primary, P < 0.09) was higher in the diestrous phase (Fig 1).

**Fig 2:**
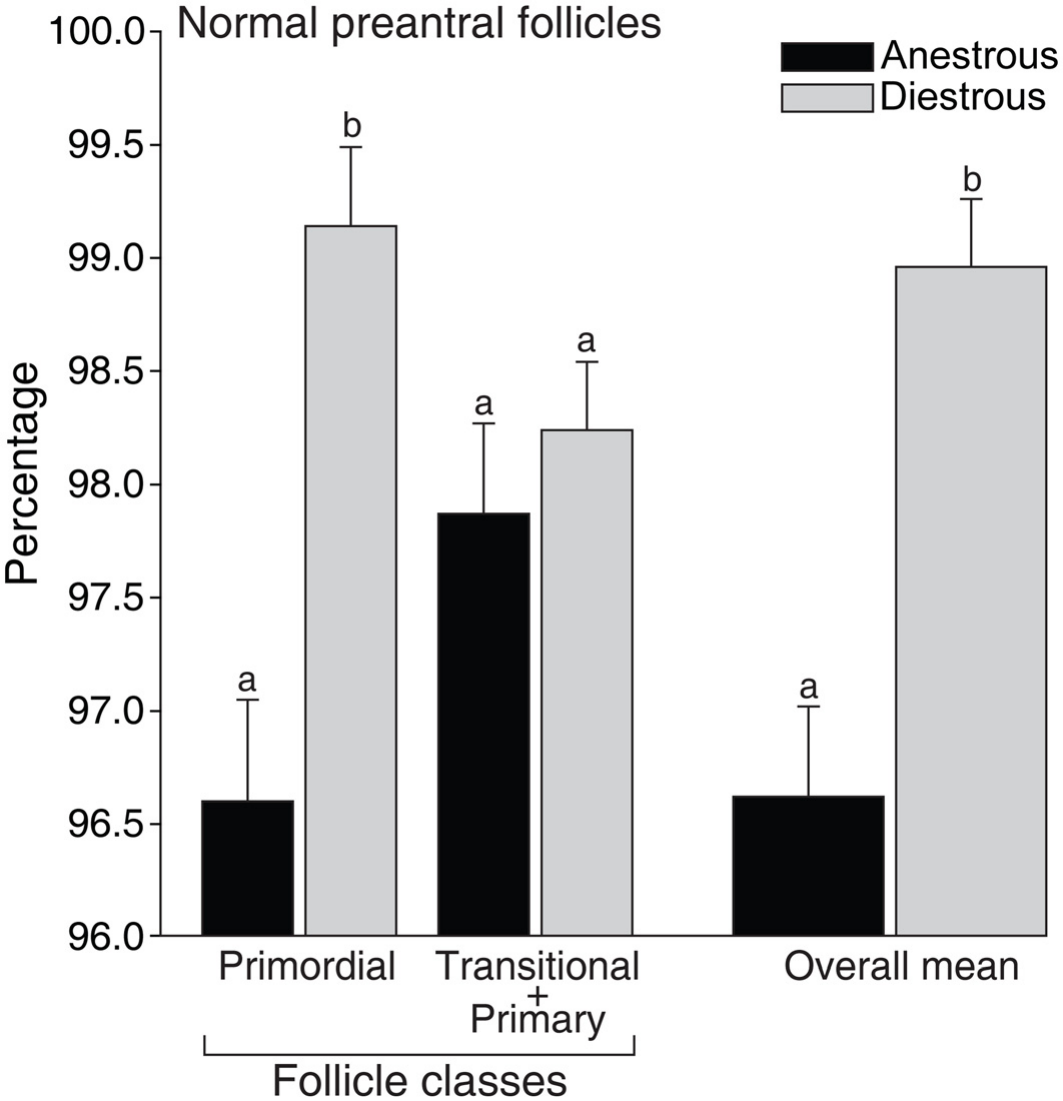
Mean (± SEM) percentage of morphologically normal equine preantral follicles according to class distribution in ovarian biopsy fragments collected during anestrous and diestrous phases. ^a,b^ Within the same follicle class and overall mean, values without a common letter differed (*P* < 0.05). Because of the low number of primary follicles (n = 5) observed in the anestrous phase, transitional and primary follicles classes were combined.

The overall percentage of normal preantral follicles differed (P < 0.05) among anestrous (96%) and diestrous phases (98%) (Fig 2). The percentage of normal primordial follicles was greater (P < 0.05) during the diestrous phase (99%); however, this pattern was not observed (P > 0.05) for growing follicles (transitional and primary follicles combined).

The percentage of normal preantral follicles in biopsy fragments collected during anestrous and diestrous phases according to the presence of different ovarian structures is shown (Table 5). Within ovaries with the same structures (follicles ≤26 mm), a greater (P < 0.05) percentage of normal preantral follicles was observed during the diestrous phase. Moreover, in the diestrous phase the percentage of normal preantral follicles tended to be higher (P < 0.09) in fragments collected from ovaries with a corpus luteum compared to ovaries with preovulatory follicles (≥36 mm). Overall, a higher (P < 0.05) preantral follicle normality rate was observed in biopsy fragments harvested from ovaries with the presence of a corpus luteum. The combination of preovulatory follicle with a corpus luteum in the same ovary was not considered for assessing the effect of ovarian structures on preantral follicle quality due to the small sample size (7.9%; n = 3/38).

**Table 5:** Percentage of morphologically normal equine preantral follicles in biopsy fragments collected during anestrous and diestrous phases, and presence of different ovarian structures (antral follicles and corpus luteum).

	Normal preantral follicles (%)		
Ovarian structures^†^	Anestrous (n = 582)^‡^	Diestrous (n = 796)	Overall (n = 1,378)
Follicles ≤26 mm	96.6^a^ (562/582)	98.8^b^^A^^B^ (352/356)	97.4^A^ (914/938)
Preovulatory follicle		97.9^A^ (96/98)	97.9^A^ (96/98)
Corpus luteum		99.7^B^^§^ (341/342)	99.7^B^^§^ (341/342)

^†^ Antral follicles ≤26 mm: 6 to 26 mm in diameter; Preovulatory follicle: ≥36 mm; and Corpus luteum: ≥30 mm, days 4 to 12 of estrous cycle.

^‡^ Total number of preantral follicles evaluated.

^a,b^ Within a row, values without a common superscript differed (P < 0.05).

^A,B^ Within a column, values without a common superscript differed (P < 0.05).

^§^ Tended to differ from the preovulatory follicle (P < 0.09).

### Effect of reproductive phase on stromal cell density

**Fig 3:**
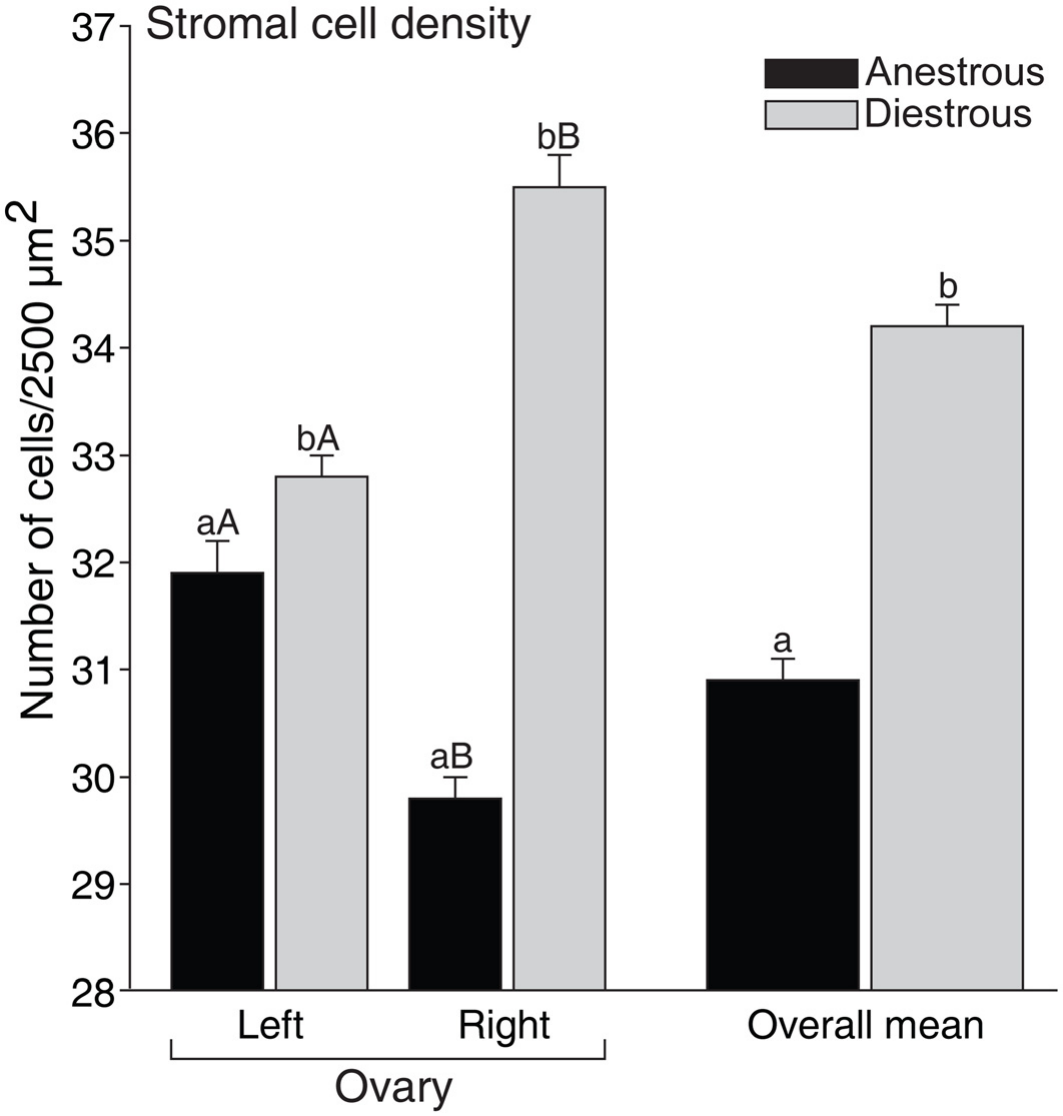
Mean (± SEM) density of equine ovarian stromal cells in biopsy fragments collected from left and right ovaries during anestrous and diestrous phases. ^a,b^ Within the same ovarian side and overall mean, values without a common letter differed (*P* < 0.05). ^A,B^ Within the same reproductive phase, left and right ovary values without a common letter differed (*P* < 0.05).

**Fig 4:**
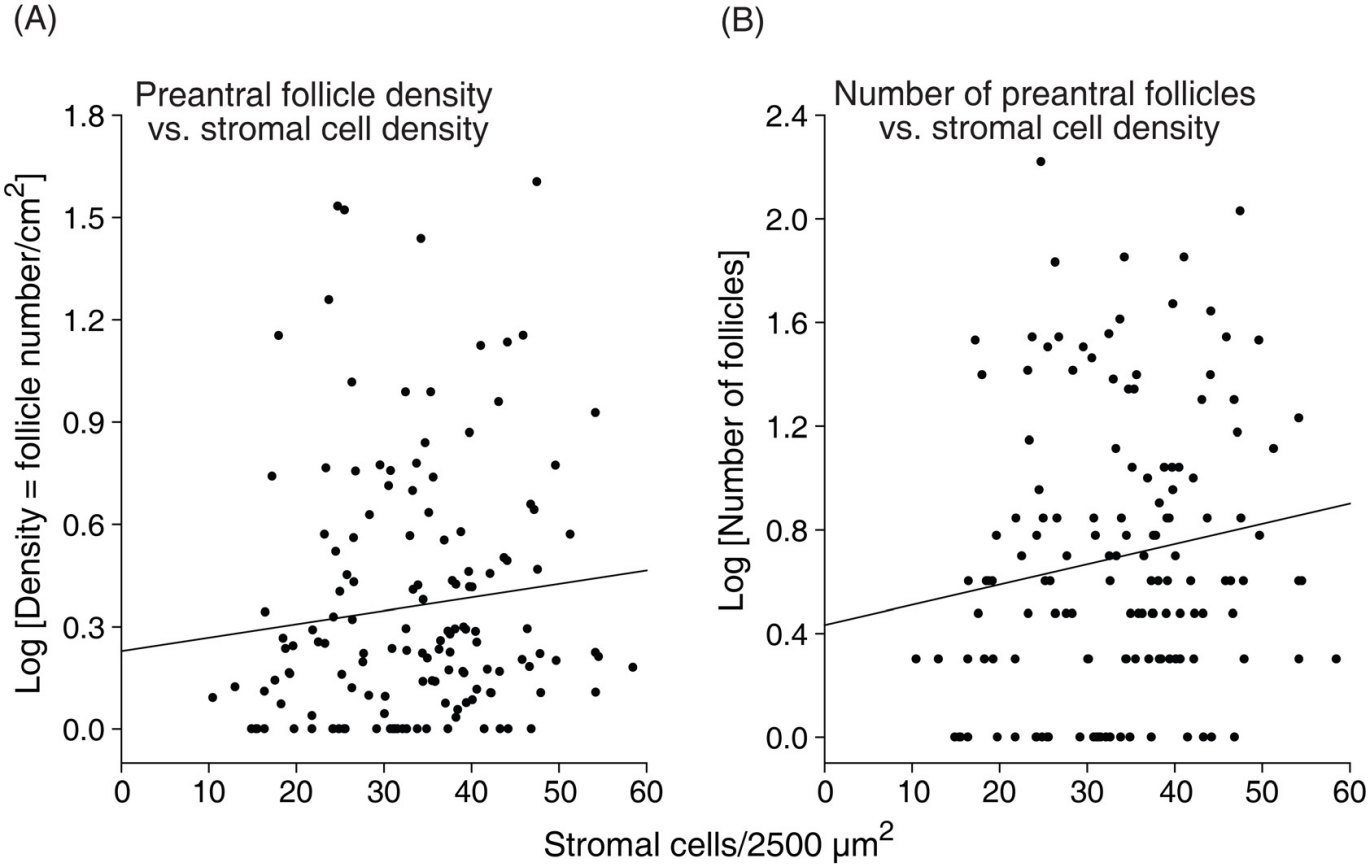
Correlation between (A) follicular density and stromal cell density, and (B) number of preantral follicles and stromal cell density. The association among variables (black line) was evaluated by Spearman correlation coefficient [(A): r = 0.15, *P* < 0.07; (B): r = 0.16, *P* < 0.05]. Each point on the chart represents one ovarian fragment evaluated (n = 142).

The mean stromal cell density (cells/2,500 μm^2^) of the ovarian fragments was 32.0 ± 0.1 cells (range, 6 to 64 cells; CV = 37%). The cell density increased (P < 0.05) in the diestrous phase (34.2 ± 0.1) when compared to the anestrous phase (30.9 ± 0.1), regardless of the ovary side (Fig 3). In addition, when comparing the ovary sides within the same reproductive phases, the left ovary in anestrous and right ovary in diestrous phases had higher (P < 0.05) stromal cell densities. Positive correlations of stromal cell density with follicle density (P < 0.07) and number of preantral follicles (P < 0.05) per ovarian fragment were observed (Fig 4A and 4B).

### Effect of ovarian structures on preantral follicle and stromal cell densities

**Fig 5:**
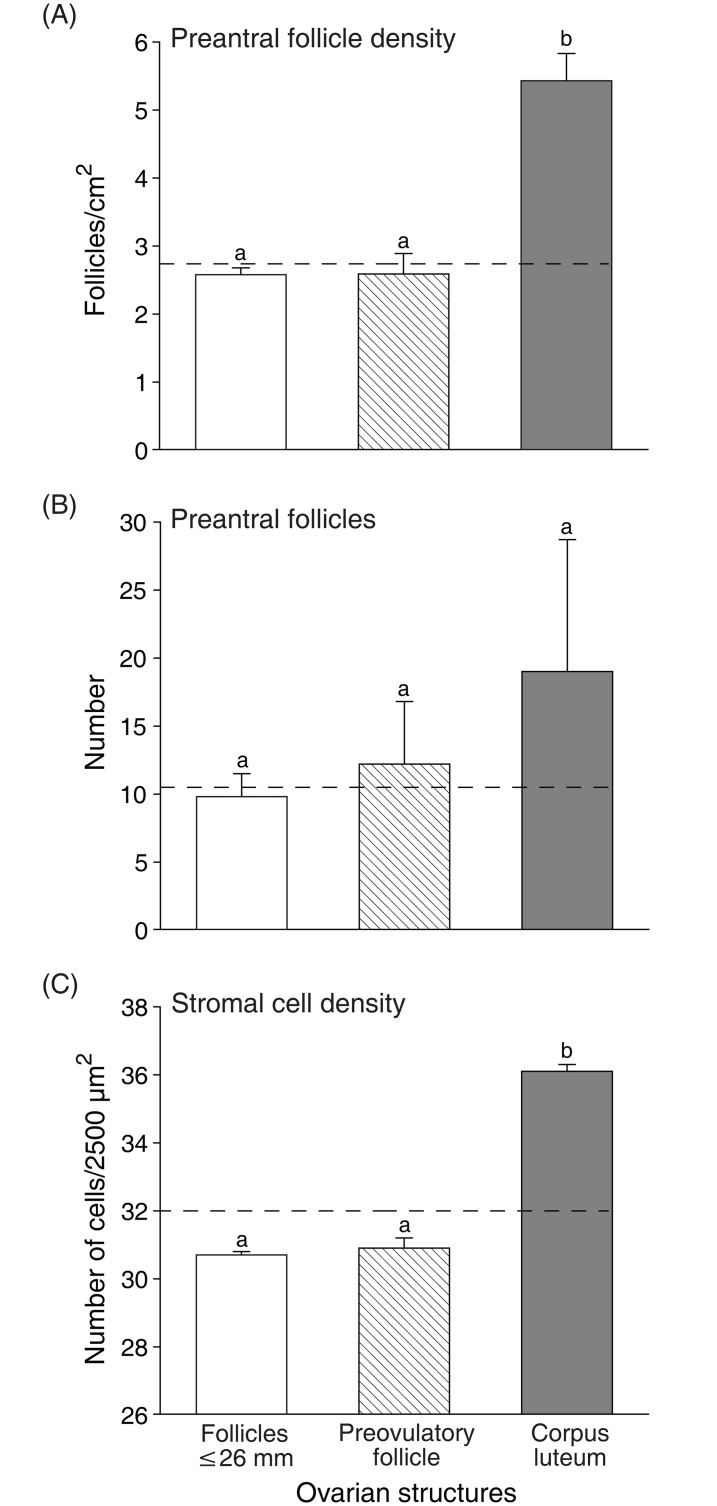
Mean (± SEM) (A) follicle density, (B) number of preantral follicles, and (C) ovarian stromal cell density in biopsy fragments collected from ovaries with antral follicles and corpus luteum. Dashed line represents the average for (A) follicle density, (B) number of preantral follicles, and (C) stromal cell density. ^a,b^ Within the same parameter evaluated, values without a common letter differed (*P* < 0.05). Follicles ≤26 mm: 6 to 26 mm in diameter; Preovulatory follicle: ≥36 mm; and Corpus luteum: ≥30 mm, days 4 to 12 of estrous cycle.

**Fig 6:**
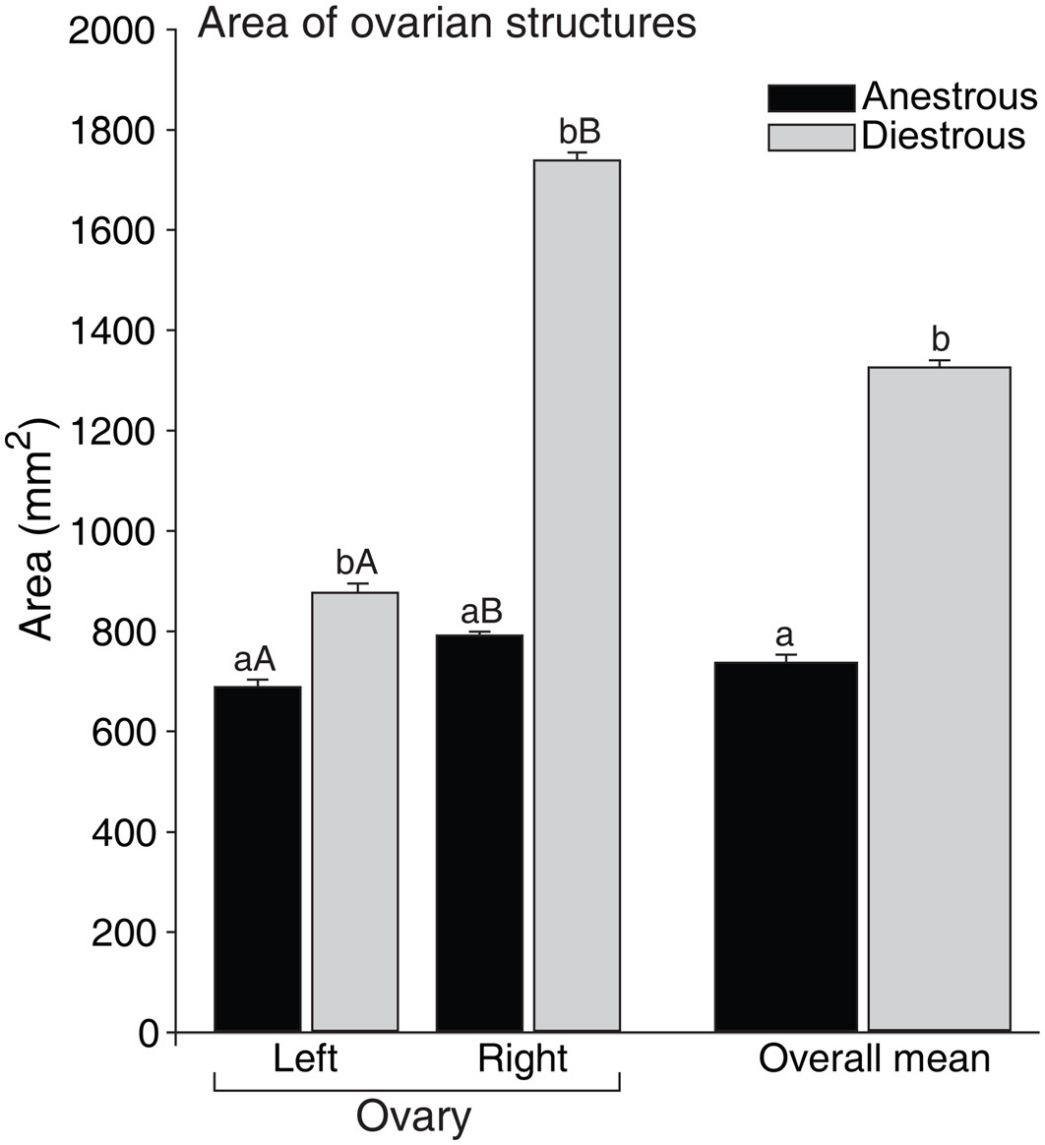
Mean (± SEM) area (mm^2^) of equine ovarian structures (antral follicles and corpus luteum) during the biopsy pick-up procedures performed during anestrous and diestrous phases. ^a,b^ Within the same ovarian side and overall mean, values without a common letter differed (*P* < 0.05). ^A,B^ Within the same reproductive phase, left and right ovary values without a common letter differed (*P* < 0.05).

**Fig 7:**
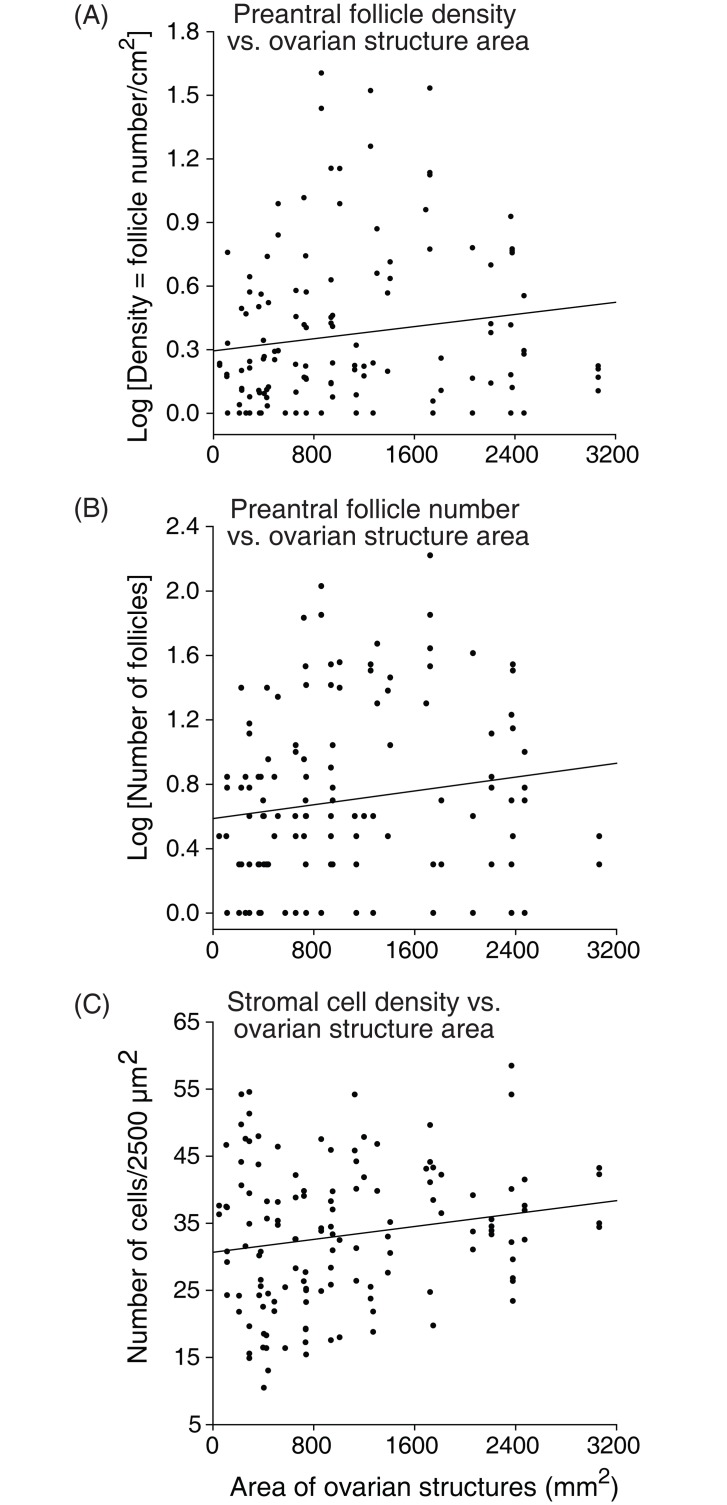
Correlation coefficients between (A) follicle density, (B) number of preantral follicles, and (C) ovarian stromal cell density with area of ovarian structures (antral follicles and corpus luteum). The association among variables (black line) was evaluated by Spearman correlation [(A): r = 0.21, (B): r = 0.21, (C): r = 0.10; *P* < 0.05]. Each point on the chart represents one ovarian fragment evaluated.

Number of preantral follicles, and follicle and stromal cell densities evaluated according to the type of ovarian structures present in the ovary are shown (Fig 5). Biopsy fragments harvested from ovaries with corpus luteum showed a greater (P < 0.05) mean follicle density (5.4 ± 0.4) when compared with fragments from ovaries with preovulatory follicles (2.5 ± 0.3), and follicles ≤26 mm (2.5 ± 0.1; Fig 5A). Nevertheless, the mean number of preantral follicles recorded was similar (P > 0.05) among fragments from ovaries with all types of structures (follicles ≤26 mm: 9.8 ± 1.7; preovulatory follicle: 12.2 ± 4.6; and corpus luteum: 19.0 ± 9.7; Fig 5B). The mean stromal cell density was similar (P > 0.05) among ovaries with follicles ≤26 mm (30.7 ± 0.1) and preovulatory follicles (30.9 ± 0.3), and differed (P < 0.05) from ovaries with corpus luteum (36.1 ± 0.2; Fig 5C). In addition, the overall mean area (mm^2^) of ovarian structures was greater (P < 0.05) in the diestrous phase and the right ovary had a greater area (P < 0.05) of ovarian structures when compared to the left ovary regardless of reproductive phase (Fig 6). Positive correlations (P < 0.05) were observed between the area of ovarian structures and follicle density, number of preantral follicles, and stromal cell density per ovarian fragment (Fig 7A–7C).

## Discussion

Studies with human ovaries face ethical barriers and limited availability of material for research, concerns which are lesser with domestic animals. Considering the similarities between women and mares relative to the dynamics of reproductive cycles [5, 7–9], age-associated changes in fertility [11], and the physiological preantral follicle heterogeneity [13], the mare can be considered an important animal model to advance knowledge regarding follicular population and density in ovarian biopsy fragments. Thus, studies with preantral follicles in mares (as an animal model) may provide relevant information that could be applied in the future on clinical human reproduction.

To our knowledge, this study reports for the first time in mares the influence of reproductive phases (anestrous and diestrous), ovary side (left and right), and ovarian structures (antral follicles and corpus luteum) on number and density of preantral follicles, and on ovarian stromal cell density from ovarian biopsy fragments.

Primordial follicles constitute the sole and critical reserve for all further follicle recruitment [35]. In this study, a higher percentage of primordial follicles was observed in the anestrous phase; however, the percentage of growing follicles (transitional and primary combined) and the overall percentage of normal follicles were greater during the diestrous phase. These results indicated that an increase in primordial follicle activation [36] occurred during the diestrous phase potentially due to higher levels of gonadotropins (FSH and LH) and several growth factors (epidermal growth factor, EGF; transforming growth factor beta, TGF-β; and vascular endothelial growth factor, VEGF) during the breeding season [37]. In addition, studies *in vitro* have shown the benefits of the aforementioned stimulatory factors on the survival and development of preantral follicles [38, 39]. A higher ovarian stromal cell density in both ovary sides was observed in the diestrous phase compared to the anestrous. Overall, a positive correlation of stromal cell density with number and density of preantral follicles was observed. Stromal cell density (number of cells per μm^2^) in mares was different when compared to bitches (1.7-fold higher; [34]), or goats and ewes (8.5-fold lower; [40]). Stromal cells are essential for the development and survival of grafted isolated follicles and have an important role in co-culture systems [41, 42]. Furthermore, theca cells are derived from stromal cells and together produce steroids (androgens) and growth factors (TGF-β, EGF, bone morphogenetic protein 7, BMP-7), which play a role in follicle activation and survival [43–46]. Once stromal cell and preantral follicle density have an important role in female fertility, the association of these factors should be considered when selecting the most suitable ovarian biopsy fragments to be used for ART programs.

In the present study, an increase in follicle density during the diestrous phase was observed when compared to the anestrous phase, regardless of the ovary side. In addition, a high disparity in follicle density among and within animals was detected in both reproductive phases. In a recent study [13] in which the reproductive phase of the estrous cycle was unknown, our group also reported a high heterogeneity in follicle density among and within mares. Contrary to the results from follicle density, the overall mean number of equine preantral follicles in biopsy fragments in the present study was similar for both ovaries (left and right) and reproductive phases (anestrous and diestrous). One possible explanation for these two findings is that the biopsies were performed in different areas of the ovarian stroma and recovered fragments with different sizes and heterogeneous follicle population [27]. On the other hand, the follicle density in biopsy fragments uses an established measure unity (area) which equalizes all samples, providing a more accurate parameter to evaluate preantral follicle population for further use in ART programs.

In our study, the biopsies were performed in different reproductive conditions (anestrous and diestrous). In order to explain the influence of ovarian structures on the number of preantral follicles and follicle and stromal cell densities, we analyzed the data in two ways: (1) total area of ovarian structures (small antral follicles, preovulatory follicle, and corpus luteum), and (2) type of ovarian structures present at the moment of the biopsy procedure. Regarding the first aspect, the overall mean area of ovarian structures increased by over 79% in the diestrous phase. In addition, the area of structures had a positive correlation with the number of preantral follicles and follicle and stromal cell densities, and the right ovary showed a greater area than the left ovary in both studied reproductive phases. Moreover, biopsy fragments collected from ovaries with a corpus luteum had greater follicle and stromal cell densities and percentage of normal preantral follicles. The influence of corpus luteum on number of preantral follicles in different species has been reported (bovine: [47]; bubaline: [23]; ovine: [21]). The primary function of the corpus luteum is the production of progesterone [48] and prostaglandin F2α [49], hormones that can improve the quality of preantral follicles [50]. Furthermore, the proliferation index of endothelial cells is intense in the early luteal phase [51], which is characterized by a dense network of capillaries in the ovary. Therefore, a potential physiological mechanism behind our findings might be that the presence of an active and highly vascularized corpus luteum may contribute to better diffusion of growth factors and hormones throughout the ovarian stroma, favoring the quality of preantral follicles. In addition, a positive correlation between the degree of cell proliferation and vascular area has been reported during early preantral follicle growth [52]. Consequently, the greater percentage of transitional and primary follicles and normal primordial follicles observed in our data during the diestrous phase makes it tempting to speculate that preantral follicle recruitment in mares may be influenced by stromal vascular supply and blood flow perfusion. Further studies need to be performed to address this subject.

**Fig 8:**
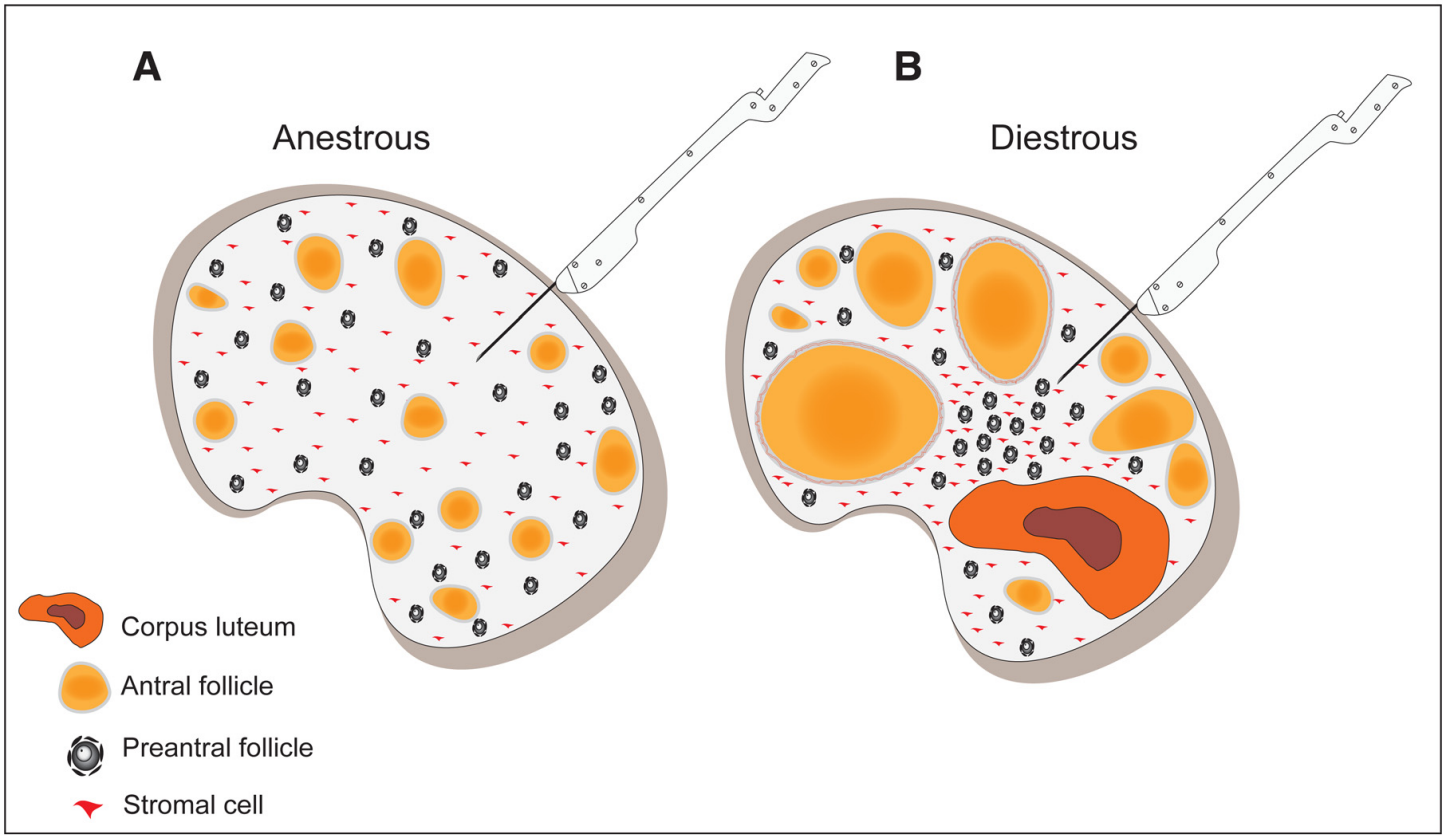
Model proposing the difference of follicle density, number of preantral follicles, and stromal cell density among anestrous (A) and diestrous (B) phases. The increase of the area of ovarian structures during the diestrous phase due to the presence of large follicle(s) and corpus luteum may increase the ovarian compression and consequently the clustering of preantral follicles and stromal cells per unit area in ovarian fragments harvested by biopsy procedure.

To our knowledge, there are no studies that described the association among the number of preantral follicles, follicle density, and stromal cell density with the area of ovarian structures for any species. This fact allowed us to propose a hypothetical mechanism to support the results observed in our study (Fig 8). During the anestrous phase in mares, there is low follicle activity and small follicles are present in the ovarian stroma. In contrast, in the diestrous phase, multiple structures such as small and medium antral follicles, preovulatory follicles, and corpus luteum can be present in the same ovary. This fact indicates that denser structures, such as the corpus luteum associated or not with larger antral follicles, can exert a greater pressure in the ovarian parenchyma and cause a consequent clustering of preantral follicles and stromal cells per unit area. In the anestrous phase, the absence of large structures allows lower compression in the ovarian tissue and therefore a possible dispersion of the preantral follicle population in the ovary.

In summary, our results indicated that: (1) the diestrous phase influenced positively the quality of preantral follicles, percentage of growing follicles, and follicle and stromal cell densities; (2) the area of ovarian structures affected the stromal cell density, and the number of preantral follicles and follicle density; and (3) the corpus luteum had a positive effect on the quality of preantral follicles, and follicle and stromal cell densities. In addition, we discovered herein a potential ideal scenario for collection of richer ovarian fragments (i.e. higher preantral follicle and stromal cell densities) using ovarian biopsy procedure. These findings reinforce the concept of the use of the mare as a model to provide comparative insights about preantral follicle density and ovarian plasticity.

## Supporting Information

S1 Table (XLS)
